# {2-Eth­oxy-6-[2-(piperidinium-1-yl)ethyl­imino­meth­yl]phenolato}diiodidozinc(II)

**DOI:** 10.1107/S1600536809048491

**Published:** 2009-11-21

**Authors:** Jing-Yan Li

**Affiliations:** a Department of Chemistry, Baicheng Normal College, Baicheng 137000, People’s Republic of China

## Abstract

The title Schiff base complex, [ZnI_2_(C_16_H_24_N_2_O_2_)], is a mononuclear zinc(II) compound. The Zn atom is four-coordinated in a distorted tetra­hedral geometry by one phenolate O atom and one imine N atom of the Schiff base ligand and by two iodide ions. In the crystal structure, mol­ecules are linked through inter­molecular N—H⋯O hydrogen bonds, forming chains running along the *a* axis.

## Related literature

For background to the applications of Schiff bases, see: Averseng *et al.* (2001 ▶); Patra *et al.* (2002 ▶); Chen *et al.* (2003 ▶); Ruck & Jacobsen (2002 ▶). For the structures of related Schiff base zinc complexes, see: Wei *et al.* (2007 ▶); Zhu, Yang *et al.* (2009 ▶); Zhu, Yin, Li *et al.* (2009 ▶); Zhu, Yin, Yang *et al.* (2009 ▶).
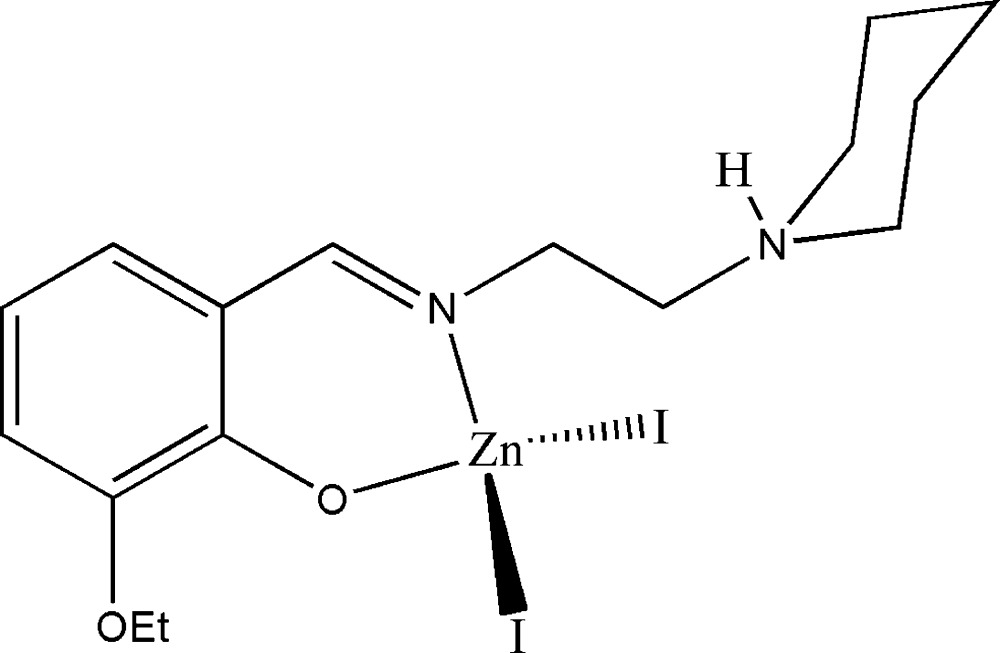


## Experimental

### 

#### Crystal data

[ZnI_2_(C_16_H_24_N_2_O_2_)]
*M* *_r_* = 595.54
Orthorhombic, 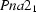

*a* = 13.5934 (10) Å
*b* = 10.2381 (8) Å
*c* = 14.7871 (11) Å
*V* = 2057.9 (3) Å^3^
*Z* = 4
Mo *K*α radiation
μ = 4.20 mm^−1^
*T* = 298 K
0.18 × 0.17 × 0.17 mm

#### Data collection

Bruker SMART CCD area-detector diffractometer
Absorption correction: multi-scan (*SADABS*; Sheldrick, 1996 ▶) *T*_min_ = 0.518, *T*_max_ = 0.535
11751 measured reflections
4438 independent reflections
3763 reflections with *I* > 2σ(*I*)
*R*_int_ = 0.029

#### Refinement

*R*[*F* ^2^ > 2σ(*F* ^2^)] = 0.028
*wR*(*F* ^2^) = 0.068
*S* = 1.02
4438 reflections
212 parameters
2 restraints
H atoms treated by a mixture of independent and constrained refinement
Δρ_max_ = 0.58 e Å^−3^
Δρ_min_ = −0.88 e Å^−3^
Absolute structure: Flack (1983 ▶), 2111 Friedel pairs
Flack parameter: 0.02 (2)

### 

Data collection: *SMART* (Bruker, 2002 ▶); cell refinement: *SAINT* (Bruker, 2002 ▶); data reduction: *SAINT*; program(s) used to solve structure: *SHELXS97* (Sheldrick, 2008 ▶); program(s) used to refine structure: *SHELXL97* (Sheldrick, 2008 ▶); molecular graphics: *SHELXTL* (Sheldrick, 2008 ▶); software used to prepare material for publication: *SHELXL97*.

## Supplementary Material

Crystal structure: contains datablocks global, I. DOI: 10.1107/S1600536809048491/sj2684sup1.cif

Structure factors: contains datablocks I. DOI: 10.1107/S1600536809048491/sj2684Isup2.hkl

Additional supplementary materials:  crystallographic information; 3D view; checkCIF report

## Figures and Tables

**Table 1:** Selected bond lengths (Å)

Zn1—O1	1.950 (2)
Zn1—N1	2.021 (3)
Zn1—I1	2.5448 (9)
Zn1—I2	2.5651 (9)

**Table 2:** Hydrogen-bond geometry (Å, °)

*D*—H⋯*A*	*D*—H	H⋯*A*	*D*⋯*A*	*D*—H⋯*A*
N2—H2⋯O2^i^	0.90 (4)	2.61 (5)	3.237 (5)	127 (4)
N2—H2⋯O1^i^	0.90 (4)	2.01 (5)	2.867 (5)	159 (5)

Symmetry code: (i) 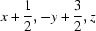.

**Fig. 1.**
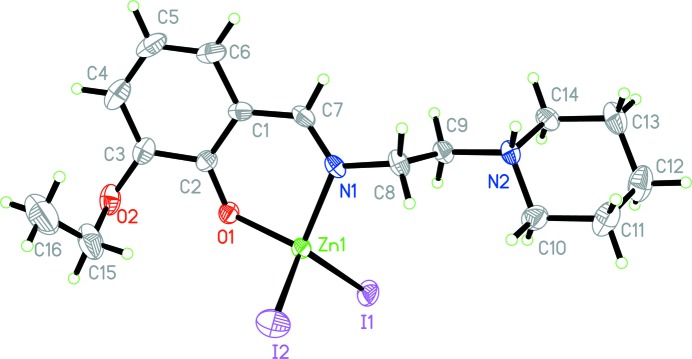
The molecular structure of the title compound, with 30% displacement ellipsoids for non-hydrogen atoms.

**Fig. 2.**
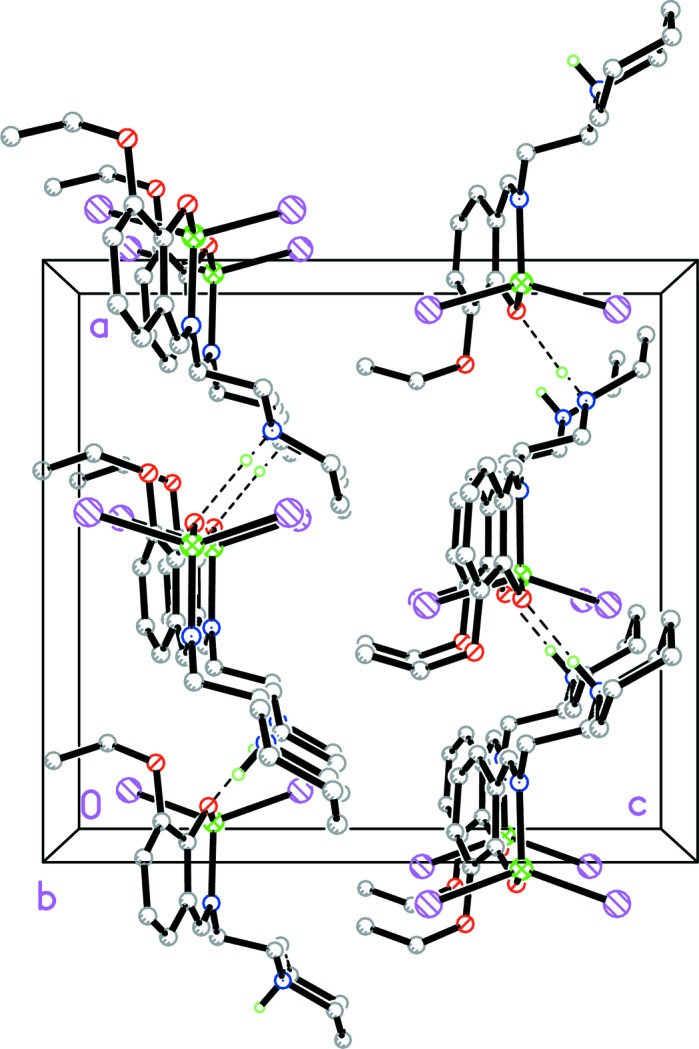
The molecular packing of the title compound, viewed along the b axis. Intermolecular hydrogen bonds are shown as dashed lines.

## supplementary crystallographic information

### Comment

Schiff bases are versatile ligands for the preparation in the preparation of metal complexes (Averseng *et al.*, 2001; Patra *et al.*, 2002; Chen *et al.*, 2003; Ruck & Jacobsen, 2002). In this paper, the new zinc(II)title complex with the Schiff base ligand 2-ethoxy-6-[(2-piperidin-1-ylethylimino)methyl]phenol is reported.

In the title complex, Fig. 1, the Zn atom is four-coordinated by one phenolate O and one imine N atoms of the Schiff base ligand, and by two iodide atoms, forming a tetrahedral coordination. The coordinate bond lengths (Table 1) and angles are comparable to those of similar zinc complexes (Wei *et al.*, 2007; Zhu, Yang *et al.*, 2009; Zhu, Yin, Li *et al.*, 2009; Zhu, Yin, Yang *et al.*, 2009).

In the crystal structure, molecules are linked through intermolecular N—H···O hydrogen bonds (Table 2), forming chains running along the *a* axis (Fig. 2).

### Experimental

2-Ethoxysalicylaldehyde (1.0 mmol, 166 mg), 2-piperidin-1-ylethylamine (1.0 mmol, 128 mg), and ZnI_2_ (1.0 mmol, 319 mg) were mixed in a methanol solution (50 ml). The mixture was stirred at reflux for 30 min to give a colourless solution. The solution was left in air for a few days, yielding colourless block-shaped crystals.

### Refinement

The H2 atom was located from a difference Fourier map and refined isotropically, with *U*_iso_ restrained to 0.08 Å^2^. Other H atoms were constrained to ideal geometries, with d(C–H) = 0.93–0.97 Å, and with *U*_iso_(H) = 1.2*U*_eq_(C), 1.5*U*_eq_(C_methyl_).

### Crystal data

**Table:**

[ZnI_2_(C_16_H_24_N_2_O_2_)]	*F*(000) = 1144
*M**_r_* = 595.54	*D*_x_ = 1.922 Mg m^−^^3^
Orthorhombic, *P**n**a*2_1_	Mo *K*α radiation, λ = 0.71073 Å
Hall symbol: P 2c -2n	Cell parameters from 4921 reflections
*a* = 13.5934 (10) Å	θ = 2.4–27.9°
*b* = 10.2381 (8) Å	µ = 4.20 mm^−^^1^
*c* = 14.7871 (11) Å	*T* = 298 K
*V* = 2057.9 (3) Å^3^	Block, colourless
*Z* = 4	0.18 × 0.17 × 0.17 mm

### Data collection

**Table:**

Bruker SMART CCD area-detector diffractometer	4438 independent reflections
Radiation source: fine-focus sealed tube	3763 reflections with *I* > 2σ(*I*)
graphite	*R*_int_ = 0.029
ω scans	θ_max_ = 27.0°, θ_min_ = 2.4°
Absorption correction: multi-scan (*SADABS*; Sheldrick, 1996)	*h* = −16→17
*T*_min_ = 0.518, *T*_max_ = 0.535	*k* = −9→13
11751 measured reflections	*l* = −18→18

### Refinement

**Table:**

Refinement on *F*^2^	Secondary atom site location: difference Fourier map
Least-squares matrix: full	Hydrogen site location: inferred from neighbouring sites
*R*[*F*^2^ > 2σ(*F*^2^)] = 0.028	H atoms treated by a mixture of independent and constrained refinement
*wR*(*F*^2^) = 0.068	*w* = 1/[σ^2^(*F*_o_^2^) + (0.0339*P*)^2^] where *P* = (*F*_o_^2^ + 2*F*_c_^2^)/3
*S* = 1.02	(Δ/σ)_max_ = 0.001
4438 reflections	Δρ_max_ = 0.58 e Å^−^^3^
212 parameters	Δρ_min_ = −0.88 e Å^−^^3^
2 restraints	Absolute structure: Flack (1983), 2111 Friedel pairs
Primary atom site location: structure-invariant direct methods	Flack parameter: 0.02 (2)

### Special details

**Table:**

Geometry. All e.s.d.'s (except the e.s.d. in the dihedral angle between two l.s. planes) are estimated using the full covariance matrix. The cell e.s.d.'s are taken into account individually in the estimation of e.s.d.'s in distances, angles and torsion angles; correlations between e.s.d.'s in cell parameters are only used when they are defined by crystal symmetry. An approximate (isotropic) treatment of cell e.s.d.'s is used for estimating e.s.d.'s involving l.s. planes.
Refinement. Refinement of *F*^2^ against ALL reflections. The weighted *R*-factor *wR* and goodness of fit *S* are based on *F*^2^, conventional *R*-factors *R* are based on *F*, with *F* set to zero for negative *F*^2^. The threshold expression of *F*^2^ > σ(*F*^2^) is used only for calculating *R*-factors(gt) *etc*. and is not relevant to the choice of reflections for refinement. *R*-factors based on *F*^2^ are statistically about twice as large as those based on *F*, and *R*- factors based on ALL data will be even larger.

### Fractional atomic coordinates and isotropic or equivalent isotropic displacement parameters (Å^2^)

**Table:**

	*x*	*y*	*z*	*U*_iso_*/*U*_eq_	
Zn1	−0.02523 (3)	0.78151 (4)	0.73753 (7)	0.03660 (10)	
I1	−0.07635 (3)	0.89935 (4)	0.881593 (19)	0.05811 (13)	
I2	−0.07707 (3)	0.89530 (5)	0.590518 (18)	0.06408 (15)	
N1	0.1209 (2)	0.7453 (3)	0.7350 (4)	0.0383 (6)	
N2	0.2829 (3)	0.9941 (4)	0.8503 (2)	0.0380 (8)	
O1	−0.06258 (15)	0.5977 (2)	0.7346 (4)	0.0386 (5)	
O2	−0.1483 (3)	0.3910 (3)	0.6564 (2)	0.0567 (9)	
C1	0.1005 (3)	0.5138 (4)	0.7039 (3)	0.0405 (10)	
C2	−0.0040 (3)	0.5075 (4)	0.7033 (3)	0.0373 (9)	
C3	−0.0463 (4)	0.3937 (4)	0.6658 (4)	0.0491 (11)	
C4	0.0100 (5)	0.2871 (5)	0.6416 (4)	0.0660 (15)	
H4	−0.0202	0.2112	0.6210	0.079*	
C5	0.1109 (5)	0.2932 (5)	0.6478 (4)	0.0677 (16)	
H5	0.1485	0.2207	0.6322	0.081*	
C6	0.1556 (4)	0.4029 (5)	0.6762 (3)	0.0549 (13)	
H6	0.2240	0.4063	0.6778	0.066*	
C7	0.1560 (3)	0.6312 (4)	0.7217 (3)	0.0415 (12)	
H7	0.2241	0.6229	0.7238	0.050*	
C8	0.1907 (3)	0.8546 (4)	0.7423 (5)	0.0457 (9)	
H8A	0.1612	0.9329	0.7174	0.055*	
H8B	0.2495	0.8349	0.7078	0.055*	
C9	0.2174 (3)	0.8774 (4)	0.8385 (3)	0.0417 (10)	
H9A	0.2506	0.8007	0.8619	0.050*	
H9B	0.1578	0.8901	0.8735	0.050*	
C10	0.2269 (4)	1.1197 (4)	0.8443 (4)	0.0549 (12)	
H10A	0.1750	1.1199	0.8893	0.066*	
H10B	0.1965	1.1265	0.7851	0.066*	
C11	0.2937 (5)	1.2366 (5)	0.8595 (4)	0.0739 (17)	
H11A	0.3412	1.2418	0.8106	0.089*	
H11B	0.2547	1.3159	0.8586	0.089*	
C12	0.3470 (5)	1.2274 (6)	0.9474 (4)	0.0751 (17)	
H12A	0.3002	1.2320	0.9968	0.090*	
H12B	0.3921	1.3003	0.9532	0.090*	
C13	0.4043 (5)	1.0991 (6)	0.9531 (5)	0.0674 (16)	
H13A	0.4553	1.0984	0.9073	0.081*	
H13B	0.4357	1.0927	1.0118	0.081*	
C14	0.3384 (4)	0.9850 (5)	0.9397 (3)	0.0504 (12)	
H14A	0.2916	0.9807	0.9891	0.060*	
H14B	0.3772	0.9055	0.9405	0.060*	
C15	−0.1804 (5)	0.4628 (8)	0.5756 (4)	0.083 (2)	
H15A	−0.2514	0.4725	0.5771	0.099*	
H15B	−0.1516	0.5495	0.5764	0.099*	
C16	−0.1523 (7)	0.3969 (10)	0.4922 (6)	0.126 (4)	
H16A	−0.0819	0.3911	0.4889	0.189*	
H16B	−0.1764	0.4456	0.4413	0.189*	
H16C	−0.1800	0.3107	0.4914	0.189*	
H2	0.333 (3)	0.987 (6)	0.811 (3)	0.080*	

### Atomic displacement parameters (Å^2^)

**Table:**

	*U*^11^	*U*^22^	*U*^33^	*U*^12^	*U*^13^	*U*^23^
Zn1	0.02854 (19)	0.0357 (2)	0.0455 (2)	−0.00197 (15)	0.0007 (4)	−0.0052 (4)
I1	0.0524 (3)	0.0591 (2)	0.0628 (3)	−0.0002 (2)	0.0065 (2)	−0.0281 (2)
I2	0.0628 (4)	0.0766 (3)	0.0528 (3)	0.0076 (2)	0.0024 (2)	0.0169 (2)
N1	0.0261 (13)	0.0474 (16)	0.0414 (15)	−0.0071 (11)	−0.003 (3)	0.002 (3)
N2	0.0350 (19)	0.0452 (19)	0.0338 (18)	−0.0077 (16)	−0.0043 (15)	−0.0033 (15)
O1	0.0316 (12)	0.0381 (13)	0.0460 (13)	−0.0049 (9)	0.007 (2)	−0.005 (2)
O2	0.059 (2)	0.053 (2)	0.058 (2)	−0.0245 (15)	0.0061 (18)	−0.0095 (16)
C1	0.040 (2)	0.045 (2)	0.037 (2)	0.0128 (19)	−0.0005 (16)	0.0015 (17)
C2	0.042 (2)	0.034 (2)	0.036 (2)	0.0008 (18)	0.0030 (16)	0.0012 (15)
C3	0.062 (3)	0.039 (3)	0.046 (3)	−0.006 (2)	0.007 (2)	−0.004 (2)
C4	0.096 (5)	0.038 (3)	0.064 (4)	−0.002 (3)	0.009 (3)	−0.006 (2)
C5	0.092 (5)	0.045 (3)	0.067 (4)	0.028 (3)	0.006 (3)	−0.009 (3)
C6	0.058 (3)	0.060 (3)	0.047 (3)	0.025 (2)	0.002 (2)	0.000 (2)
C7	0.0279 (18)	0.061 (2)	0.036 (3)	0.0033 (16)	−0.0049 (18)	−0.004 (2)
C8	0.0343 (18)	0.055 (2)	0.048 (2)	−0.0151 (16)	−0.001 (3)	0.001 (4)
C9	0.037 (2)	0.044 (2)	0.045 (2)	−0.0062 (19)	−0.0032 (19)	0.0025 (18)
C10	0.067 (3)	0.049 (3)	0.049 (3)	0.008 (2)	−0.011 (3)	−0.002 (2)
C11	0.118 (5)	0.046 (3)	0.058 (3)	−0.005 (3)	−0.015 (3)	−0.002 (2)
C12	0.110 (5)	0.059 (3)	0.056 (3)	−0.020 (3)	−0.014 (3)	−0.007 (3)
C13	0.058 (4)	0.084 (4)	0.060 (4)	−0.019 (3)	−0.014 (3)	−0.012 (3)
C14	0.047 (3)	0.059 (3)	0.046 (3)	0.002 (2)	−0.017 (2)	0.000 (2)
C15	0.050 (4)	0.122 (6)	0.076 (4)	−0.021 (4)	0.002 (3)	−0.030 (4)
C16	0.073 (5)	0.214 (12)	0.091 (5)	0.007 (6)	−0.018 (5)	−0.051 (6)

### Geometric parameters (Å, °)

**Table:**

Zn1—O1	1.950 (2)	C8—H8A	0.9700
Zn1—N1	2.021 (3)	C8—H8B	0.9700
Zn1—I1	2.5448 (9)	C9—H9A	0.9700
Zn1—I2	2.5651 (9)	C9—H9B	0.9700
N1—C7	1.277 (5)	C10—C11	1.520 (7)
N1—C8	1.471 (4)	C10—H10A	0.9700
N2—C10	1.498 (6)	C10—H10B	0.9700
N2—C9	1.500 (5)	C11—C12	1.491 (8)
N2—C14	1.524 (5)	C11—H11A	0.9700
N2—H2	0.90 (4)	C11—H11B	0.9700
O1—C2	1.304 (5)	C12—C13	1.529 (8)
O2—C3	1.394 (6)	C12—H12A	0.9700
O2—C15	1.470 (8)	C12—H12B	0.9700
C1—C6	1.421 (6)	C13—C14	1.485 (7)
C1—C2	1.421 (6)	C13—H13A	0.9700
C1—C7	1.444 (6)	C13—H13B	0.9700
C2—C3	1.412 (6)	C14—H14A	0.9700
C3—C4	1.381 (7)	C14—H14B	0.9700
C4—C5	1.376 (9)	C15—C16	1.457 (9)
C4—H4	0.9300	C15—H15A	0.9700
C5—C6	1.345 (7)	C15—H15B	0.9700
C5—H5	0.9300	C16—H16A	0.9600
C6—H6	0.9300	C16—H16B	0.9600
C7—H7	0.9300	C16—H16C	0.9600
C8—C9	1.486 (8)		
			
O1—Zn1—N1	94.50 (10)	N2—C9—H9A	109.1
O1—Zn1—I1	113.89 (16)	C8—C9—H9B	109.1
N1—Zn1—I1	111.78 (16)	N2—C9—H9B	109.1
O1—Zn1—I2	110.37 (17)	H9A—C9—H9B	107.8
N1—Zn1—I2	109.73 (17)	N2—C10—C11	111.3 (4)
I1—Zn1—I2	114.776 (17)	N2—C10—H10A	109.4
C7—N1—C8	117.8 (3)	C11—C10—H10A	109.4
C7—N1—Zn1	122.5 (3)	N2—C10—H10B	109.4
C8—N1—Zn1	119.5 (2)	C11—C10—H10B	109.4
C10—N2—C9	112.0 (3)	H10A—C10—H10B	108.0
C10—N2—C14	110.8 (4)	C12—C11—C10	111.7 (5)
C9—N2—C14	110.3 (3)	C12—C11—H11A	109.3
C10—N2—H2	115 (4)	C10—C11—H11A	109.3
C9—N2—H2	108 (4)	C12—C11—H11B	109.3
C14—N2—H2	101 (4)	C10—C11—H11B	109.3
C2—O1—Zn1	122.1 (2)	H11A—C11—H11B	107.9
C3—O2—C15	111.5 (4)	C11—C12—C13	110.5 (5)
C6—C1—C2	119.3 (4)	C11—C12—H12A	109.6
C6—C1—C7	116.2 (4)	C13—C12—H12A	109.6
C2—C1—C7	124.1 (4)	C11—C12—H12B	109.6
O1—C2—C3	118.4 (4)	C13—C12—H12B	109.6
O1—C2—C1	125.1 (4)	H12A—C12—H12B	108.1
C3—C2—C1	116.5 (4)	C14—C13—C12	111.2 (5)
C4—C3—O2	120.7 (4)	C14—C13—H13A	109.4
C4—C3—C2	121.9 (5)	C12—C13—H13A	109.4
O2—C3—C2	117.4 (4)	C14—C13—H13B	109.4
C5—C4—C3	120.0 (5)	C12—C13—H13B	109.4
C5—C4—H4	120.0	H13A—C13—H13B	108.0
C3—C4—H4	120.0	C13—C14—N2	111.5 (4)
C6—C5—C4	120.6 (5)	C13—C14—H14A	109.3
C6—C5—H5	119.7	N2—C14—H14A	109.3
C4—C5—H5	119.7	C13—C14—H14B	109.3
C5—C6—C1	121.3 (5)	N2—C14—H14B	109.3
C5—C6—H6	119.4	H14A—C14—H14B	108.0
C1—C6—H6	119.4	C16—C15—O2	112.3 (7)
N1—C7—C1	126.5 (4)	C16—C15—H15A	109.1
N1—C7—H7	116.8	O2—C15—H15A	109.1
C1—C7—H7	116.8	C16—C15—H15B	109.1
N1—C8—C9	110.4 (5)	O2—C15—H15B	109.1
N1—C8—H8A	109.6	H15A—C15—H15B	107.9
C9—C8—H8A	109.6	C15—C16—H16A	109.5
N1—C8—H8B	109.6	C15—C16—H16B	109.5
C9—C8—H8B	109.6	H16A—C16—H16B	109.5
H8A—C8—H8B	108.1	C15—C16—H16C	109.5
C8—C9—N2	112.5 (3)	H16A—C16—H16C	109.5
C8—C9—H9A	109.1	H16B—C16—H16C	109.5

### Hydrogen-bond geometry (Å, °)

**Table:**

*D*—H···*A*	*D*—H	H···*A*	*D*···*A*	*D*—H···*A*
N2—H2···O2^i^	0.90 (4)	2.61 (5)	3.237 (5)	127 (4)
N2—H2···O1^i^	0.90 (4)	2.01 (5)	2.867 (5)	159 (5)

Symmetry codes: (i) *x*+1/2, −*y*+3/2, *z*.

## References

[bb1] Averseng, F., Lacroix, P. G., Malfant, I., Périssé, N. & Lepetit, C. (2001). *Inorg. Chem.* **40**, 3797-3804. 10.1021/ic0013429 11442379

[bb2] Bruker (2002). *SAINT* and *SMART*. Bruker AXS Inc., Madison, Wisconsin, USA.

[bb3] Chen, C., Huang, D., Zhang, X., Chen, F., Zhu, H., Liu, Q., Zhang, C., Liao, D., Li, L. & Sun, L. (2003). *Inorg. Chem.* **42**, 3540–3548. 10.1021/ic025944z 12767191

[bb4] Flack, H. D. (1983). *Acta Cryst.* A**39**, 876–881.

[bb5] Patra, A. K., Olmstead, M. M. & Mascharak, P. K. (2002). *Inorg. Chem.* **41**, 5403–5409. 10.1021/ic020373w 12377034

[bb6] Ruck, R. T. & Jacobsen, E. N. (2002). *J. Am. Chem. Soc.* **124**, 2882–2883. 10.1021/ja025588j 11902873

[bb7] Sheldrick, G. M. (1996). *SADABS*. University of Göttingen, Germany.

[bb8] Sheldrick, G. M. (2008). *Acta Cryst.* A**64**, 112–122. 10.1107/S0108767307043930 18156677

[bb9] Wei, Y.-J., Wang, F.-W. & Zhu, Q.-Y. (2007). *Acta Cryst.* E**63**, m654–m655.

[bb10] Zhu, X.-W., Yang, X.-Z., Zhang, C.-X., Li, G.-S. & Yin, Z.-G. (2009). *Acta Cryst.* E**65**, m1332–m1333. 10.1107/S1600536809040495 PMC2971035 21578090

[bb11] Zhu, X.-W., Yin, Z.-G., Li, G.-S., Yang, X.-Z. & Zhang, C.-X. (2009). *Acta Cryst.* E**65**, m1226–m1227. 10.1107/S1600536809037210 PMC2970395 21577747

[bb12] Zhu, X.-W., Yin, Z.-G., Yang, X.-Z., Li, G.-S. & Zhang, C.-X. (2009). *Acta Cryst.* E**65**, m1293–m1294. 10.1107/S1600536809038446 PMC2971364 21578061

